# Everybody's Page

**Published:** 1888-05-19

**Authors:** 


### Everybody's Page.

In the ventilation of any room means should be provided both for the ingress of pure air and the egress of the foul air. A single opening at the roof or the floor can at best provide only very defective ventilation.

M. Broca places the lowest limit of brain-weight compatible with human intelligence at 37 oz. in males, and 32 in females; the average brain-weight of Europeans being about 49 oz.

The effects of cold on the functions of the nervous system were never more strikingly displayed than among the soldiers of the first Napoleon in the disastrous retreat from Moscow in 1812, when, we are told, some of them became mad, many were seized with convulsions, others staggered about as if intoxicated, and sank into a stupor from which they could not be aroused.

It is said that cancer is increasing. This has been accounted for in several ways, but possibly one item in the explanation is that many individuals who in previous years would have died about 25 or 30 from consumption, now, owing to better surroundings, get over this danger and die later on of cancer. The increase in this disease may mean a general improvement rather than the reverse.

Trousseau, the great French physician, says: "Rickets is never so common as it is in babies weaned ere the teething is forward enough, and brought up on pap, vegetables, or even meat." Beef-tea, which is so largely used in the treatment of many fevers, if prepared in the ordinary way and cooked, is said to be four times less nutritious than milk; while raw beef juice is only equal to milk in the nutritive scale.

The stories of tea being adulterated with the leaves of the sloe, hawthorn, &c., however true they may have been at one time, seem altogether destitute of foundation in the present day. In an old Act of Parliament, however, passed in George III.'s time, we read that "great quantities of sloe leaves, and ash and elder and other trees and shrubs, were being manufactured and sold as tea, to the injury and destruction of great quantities of timber woods and underwoods.

Wherever the crowding is great and the occupation sedentary there consumption is most prevalent. Mechanics, watchmakers, compositors, printers, clerks are very prone to it; and Dr. Guy showed very clearly the influence of physical work on consumption, for he proved that in London printing establishments, of compositors and pressmen, the former were more prone to the disease. Both work in the same heated impure atmosphere, but whilst the former had little physical work to do, the latter (pressmen) had every now and then to perform work requiring a good deal of physical exertion. Even this little seems to have rendered them less liable to consumption.

When the growth is good the average length of hair on the female head will be found to vary from 22 inches to 28 inches. Anything beyond this must be regarded as exceptional. Cases, it is true, are recorded in which it has measured from five to six feet, but these are very rare. When compared, however, with the hair growth of the North American Indians, even this falls very far short. A chief of the Crows is mentioned by Catlin as possessing hair of the almost incredible length of 10 feet 7 inches. It is only on occasions of ceremony that an American Indian allows his hair to hang exposed; as a rule, it is carefully rolled round with a leathern strap, and when thus done up it weighs several pounds.

The English nation drank about £2,000,000 worth more alcohol in 1887 than in 1886, the increase being chiefly in beer.

According to Suidas, who lived in the tenth century, the Athenian ladies became very sensitive to their inferiority to man in the matter of hair, and tried hard to cultivate it on the cheek and chin. In many cases they went so far as to wear false beards.

The following is a good recipe for violet copying-ink: Dissolve forty parts of extract of logwood, five of oxalic acid, and thirty parts of sulphate of aluminum, without heat, in 800 parts of distilled water and ten parts of glycerine; let stand twenty-four hours, then add a solution of five parts of bichromate of potassium in 100 parts of distilled water, and again set aside for twenty-four hours. Now raise the mixture to the boiling point in a bright copper boiler, mix with it (while hot) fifty parts of wood vinegar, and when cold put into bottles. After a fortnight decant it from the sediment. In thin layers this ink is reddish violet; it writes dark violet, and furnishes bluish violet copies.

A new accusation has been brought against our pet beverage, tea, which we love with a tenderness second only to that felt for it by the Russians and the Chinese. Dr. W. S. Black writes to the *British Medical Journal* that he has noted an intimate connexion between tea-drinking and toothache. Tea causes inflammation and finally abscess of the fang of the tooth, with dentalgia—that is, toothache, at every stage of the disease. This is a dilemma! What are the women of England to do—give up tea or submit to toothache? The case of the old toper, who had to choose between gout and the renunciation of port, is the only one comparable with it; and probably the final choice will be the same. Ladies will cling to their beverage and risk the consequences.

The vintners of the eighteenth century were suspected, and not without reason, if we are to believe Addison, of supplying the public with liquor, which, according to the phraseology of our present Food Adulteration Act, was "not of the substance, nature, and quality of the article demanded." In a half-humorous strain the genial writer of the "Spectator" thus refers to the attempts of these subtle philosophers, as he calls them, to manufacture wine without having recourse to grape juice. He says, "They are daily employed in the transmutation of liquors, and by the power of magical drugs and incantations raise under the streets of London the choicest products of the hills and valleys of France. They squeeze Bordeaux out of a sloe and draw champagne from an apple."

Twenty years ago no one knew of the association between pulmonary consumption and a damp subsoil, but statistics have fully proved the connexion. In fifteen English towns recorded by Mr. Simon the deaths from consumption fell immediately when the subsoil was dried by a system of drainage. In Salisbury the deaths from consumption fell 49 per cent.; in Ely, 47 per cent.; and Merthyr Tydvil, which gained least, had its death-rate from consumption lowered by 11 per cent. By statistics we were pointed to the high mortality from consumption in the British army, and especially in the Guards, due to confined air—a mortality which has been so affected by better ventilation of barracks that the consumptive death-rate fell in the Guards from 125 in 10,000 in the year 1858 to 16·9 in the year 1875; that is to say, the deaths from consumption alone in the Guards in 1875 was less than a seventh of the number in 1858.

